# A Carbonized Zeolite/Chitosan Composite as an Adsorbent for Copper (II) and Chromium (VI) Removal from Water

**DOI:** 10.3390/ma16062532

**Published:** 2023-03-22

**Authors:** Endar Hidayat, Tomoyuki Yoshino, Seiichiro Yonemura, Yoshiharu Mitoma, Hiroyuki Harada

**Affiliations:** 1 Graduate School of Comprehensive and Scientific Research, Prefectural University of Hiroshima, Shobara 727-0023, Japan; hidayatendar1@gmail.com (E.H.); yoshino@pu-hiroshima.ac.jp (T.Y.); yone@pu-hiroshima.ac.jp (S.Y.); mitomay@pu-hiroshima.ac.jp (Y.M.); 2 Department of Life and Environmental Science, Prefectural University of Hiroshima, Shobara 727-0023, Japan

**Keywords:** adsorption, chitosan, Cu(II) removal, Cr(VI) removal, isotherm studies, kinetic studies, carbonized zeolite/chitosan, zeolite

## Abstract

To address Cu(II) and Cr(VI) water pollution, a carbonized zeolite/chitosan (C-ZLCH) composite adsorbent was produced via pyrolysis at 500 °C for two hours. C-ZLCH was characterized using scanning electron microscopy (SEM), energy-dispersive spectroscopy (EDS), Fourier transform infrared spectroscopy (FTIR), dynamic light scattering (DLS), and zeta potential measurements. The batch experiments were performed by varying the initial pH, concentration, and contact time. The optimal pH values for Cu(II) and Cr(VI) were 8.1 and 9.6, respectively. The highest adsorption capacities for Cu(II) and Cr(VI) were 111.35 mg/g at 60 min and 104.75 mg/g at 90 min, respectively. The effects of chemicals such as sodium (Na^+^), glucose, ammonium (NH_4_^+^), and acid red 88 (AR88) were also studied. Statistical analysis showed that sodium had no significant effect on Cu(II) removal, in contrast to Cr(VI) removal. However, there was a significant effect of the presence of glucose, ammonium, and AR88 on both Cu(II) and Cr(VI) removal. The adsorption isotherm and kinetic models were fitted using Langmuir and pseudo-second-order models for Cu(II) and Cr(VI), respectively.

## 1. Background

The aquatic environment is constantly polluted due to population increases and human activities such as industrialization [1]. Heavy metal contamination, a primary environmental concern, is mainly generated by industrial effluents (electroplating, metal finishing, ceramic, and textile industries), including Cu(II) and Cr(VI). Heavy metal ion decontamination is challenging because of its environmental stability and non-biodegradability. Long-term exposure to toxic heavy metals may cause serious health issues, including brain impairment, anemia, bone abnormalities, and cancer [2]. Therefore, practical approaches for capturing heavy metal pollution are urgently required. Researchers have thoroughly investigated heavy metal removal technologies such as ion exchange [3], chemical precipitation, reduction, electrochemical [4], adsorption [5,6,7], and membrane separation [8,9]. Of these, adsorption is among the best strategies because it is simple to use, convenient, and inexpensive [10].

A wide range of adsorbent materials, including activated carbon from coconuts [11], olive stones [12], silica composites [13], and African palm fruits [14], have been used to remove heavy metals from water. As a result, research has concentrated on developing biodegradable and natural adsorbents such as chitosan [15]. Chitosan is a cationic biopolymer formed by deacetylation of chitin, the second most prevalent polymer [16]. However, it has some disadvantages including poor recovery, low mechanical strength, swelling, and chemical resistance [17]. Furthermore, because it may dissolve in acidic solutions, chitosan is particularly sensitive to pH, which restricts its application because of the excessive protonation of its amino groups, thereby reducing its adequate adsorption capacities [18]. Cross-linking compounds such as epichlorohydrin [19,20,21], attapulgite [22], and glutaraldehyde [23] have been utilized to address these issues.

Zeolites are hydrated aluminosilicate minerals with micropores and strong mechanical resistance, which can be utilized to support the chitosan structure. They were constructed using an interconnected tetrahedral alumina (AlO_4_) structure and silica (SiO_4_). Some authors have used chitosan/zeolite composites to effectively eliminate pollutants, such as dyes [24,25], ammonium [16], fluoride [26], and heavy metals [27]. 

However, some researchers have succeeded in improving the mechanical strength of chitosan using hydrothermal [28] and pyrolysis [29] methods. However, pyrolysis is the best option because of its zero waste, high carbon yield, and reduced emissions during processing. In the hydrothermal method, processing takes a long time, has a low carbon yield, is costly to autoclave, and retains the residue during processing. Based on the aforementioned results, the carbonization of zeolite/chitosan (C-ZLCH) by pyrolysis is an appropriate solution to overcome the stability problem of chitosan. Scanning electron microscopy (SEM), energy-dispersive spectroscopy (EDS), Fourier transform infrared spectroscopy (FTIR), dynamic light scattering (DLS), and zeta potential measurements were used to characterize the C-ZLCH. The removal of Cu(II) and Cr(VI) from the water was investigated using adsorption isotherms and kinetics. Cu(II) and Cr(VI) removal was examined in the presence of coexisting chemical substances (organic and inorganic), such as sodium (Na^+^), glucose, ammonium (NH_4_^+^), and acid red 88 (AR88) dye.

## 2. Materials and Methods

### 2.1. Materials and Chemicals

Zeolite (ZL) and chitosan (CH) were supplied by Tosoh Co., Ltd., 4560 Kaisei-Cho, Shunan City, Yamaguchi Prefecture, 746-8501, Japan, and Acros Organics, Belgium, respectively. Sodium hydroxide (NaOH), acetic acid (CH_3_COOH), sodium chloride (NaCl), glucose, ammonium chloride (NH_4_Cl), potassium dichromate (K_2_Cr_2_O_7_), copper solution (1000 mg/L), hydrochloric acid (HCl), and Acid Red 88 (AR88) were purchased from Kanto Chemical Co., Inc., Tokyo, Japan.

### 2.2. Synthesis of C-ZLCH

Chitosan (1 g) was mixed with 100 mL of 1% CH_3_COOH for 24 h at ambient temperature with a magnetic stirrer (Mag-mixer MG600) (solution A). Solution A (25 mL) was mixed with the zeolite (0.5 g) for two hours at 30 °C. The mixture was incubated for 30 min with 1 M NaOH (25 mL). The mixture was filtered (qualitative paper filter no. 5C), and dried at 60 °C for 48 h (ZLCH). ZLCH was then carbonized in a muffle furnace (FO100, Yamato, Japan) at 500 °C for two hours and cooled in a desiccator for 24 h. The mixture was then ground and sieved. This adsorbent is called C-ZLCH.

### 2.3. Batch Adsorption Experiments

The studies of the adsorption of Cu(II) and Cr(VI) ions were conducted three times, and average results appeared in the figure with a bio-shaker (V-BR-36) at 30 °C. The effects of pH (2.0, 4.3, 6.7, 8.1, and 9.6), initial metal ion [Cu(II) and Cr(VI)] concentrations (10, 15, 20, and 25 mg/L), and contact time (30, 60, 90, 150, 180, 1020, and 1440 min) were evaluated. The adsorbed amount and percent adsorption were calculated using Equations (1) and (2), respectively.

(1) $$m_{/\mathrm{Mt}}=\frac{m_{0}-m_{e}}{W}V$$

(2) $$\mathrm{Percent}\;\mathrm{adsorption}\;=\frac{m_{o}-m_{e}}{m_{o}}\;\times 100$$

where

m is the adsorption capacities (mg/g),
M_t_ is the adsorption capacities at the time (mg/g),
Percent adsorption is the metal ions (Cu(II) and Cr(VI)) removal efficiency (%),
m_o_ is the initial metal concentration (mg/L),
m_e_ is the metal ion equilibrium at the time (mg/L),
W is the adsorbent weight (C-ZLCH) (g), and
V is the volume solution in the batch (L).

### 2.4. Characterization

Cu(II) and Cr(VI) ions were determined using a heavy metal test kit with a spectrophotometer (Kyoritsu Chemical-Check Lab., Corp, Yokohama, Japan). The zeta potential was measured using Zetasizer Ver. 7.13, Malvern Panalytical Ltd., Kobe, Japan. The SEM images and elemental distribution of C-ZLCH were analyzed using SEM-EDS (JIED-2300, Shimadzu, Kyoto, Japan). The functional groups of C-ZLCH before and after Cu(II) and Cr(VI) adsorption were analyzed using ATR-FTIR (Thermo Scientific Nicolet iS10, Waltham, MA, USA). Particle size distribution was measured using dynamic light scattering (DLS) (Horiba LB-550, Horiba Advanced Techno, Co., Ltd, Kyoto, Japan). DLS and FTIR spectra were processed using Origin 2022b.

### 2.5. Statistical Analysis

All the results were documented using Microsoft Excel. The effects of chemicals and dyes on Cu(II) and Cr(VI) removal were examined using a completely randomized design (CRD). Data were analyzed using ANOVA with Tukey’s test (*p* ≤ 0.05) using Minitab 21.3.1.

## 3. Results and Discussion

### 3.1. Characteristics of the Adsorbent (C-ZLCH)

**Figure 1:**
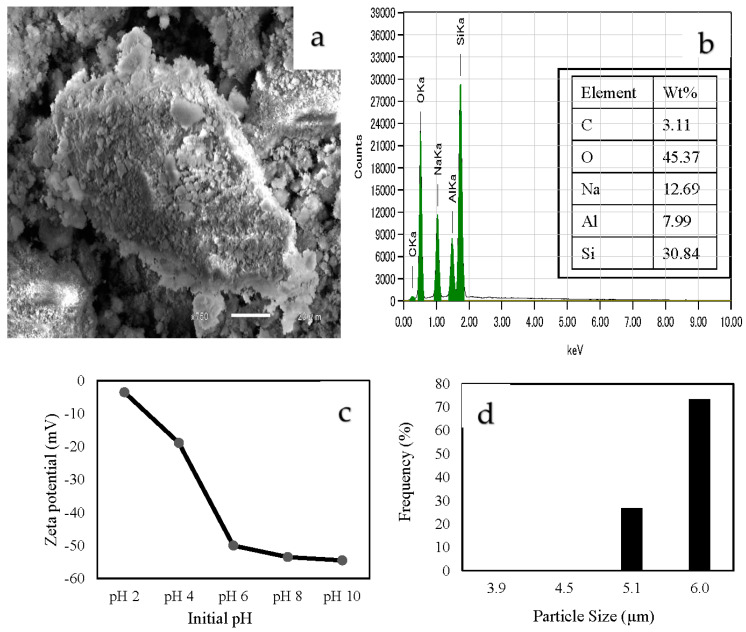
Characterization of C-ZLCH. (**a**) SEM photograph. (**b**) EDS spectra. (**c**) Zeta potential. (**d**) Particle size distribution.

Figure 1a,b show the SEM images and EDS spectra of C-ZLCH, respectively. As can be seen that the surface has a rough texture and adhesive surface. The EDS data showed that aluminum and silica were present in the zeolite material at 7.99 wt% and 30.84 wt%, respectively. However, sodium (12.69 wt%) appeared because of the alkaline-treated adsorbent preparation using sodium hydroxide. Figure 1c shows that the surface of C-ZLCH was negatively charged at all pH values from 2 to 10, with an average size of 5.5 µm (Figure 1d). 

### 3.2. FTIR Spectra of C-ZLCH before and after Cu(II) and Cr(VI) Adsorption

**Figure 2:**
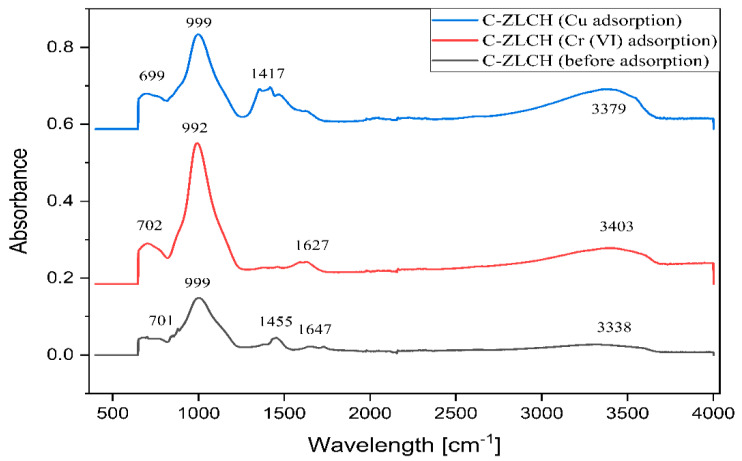
FTIR spectra of C-ZLCH before and after Cu(II) and Cr(VI) adsorption.

The FTIR data for C-ZLCH before and after Cu(II) and Cr(VI) adsorption are shown in Figure 2. The adsorption peaks at 3338 cm^−1^ increased after Cu(II) and Cr(VI) adsorption at 3379 and 3403 cm^−1^, respectively. This indicates that the stretching vibrations of –OH and –NH from chitosan interact with the metal ions [5]. A decrease peak occurred after the Cr(VI) adsorption process from 1647 to 1627 cm^−1^, which corresponds to carboxylic groups [30], and disappeared after the Cu(II) adsorption process, because carboxyl in acetate species easily decomposes when reacted on copper even at room temperature to evolve CO_2_ as in the thermal reaction [31]. A decrease in the peak intensity after Cu(II) adsorption from 1455 to 1417 cm^−1^ suggests chemical interactions between copper and and C–H bending vibrations [32]. The peaks at 999 and 992 cm^−1^ correspond to Si-O-Si or Al-O-Al [25]. The peaks at 702 cm^−1^, 701 cm^−1^, and 699 cm^−1^ correspond to the asymmetric vibrations of the Si-O (bridging) and Si-O- (non-bridging) bonds [33].

### 3.3. Initial pH Effects

**Figure 3:**
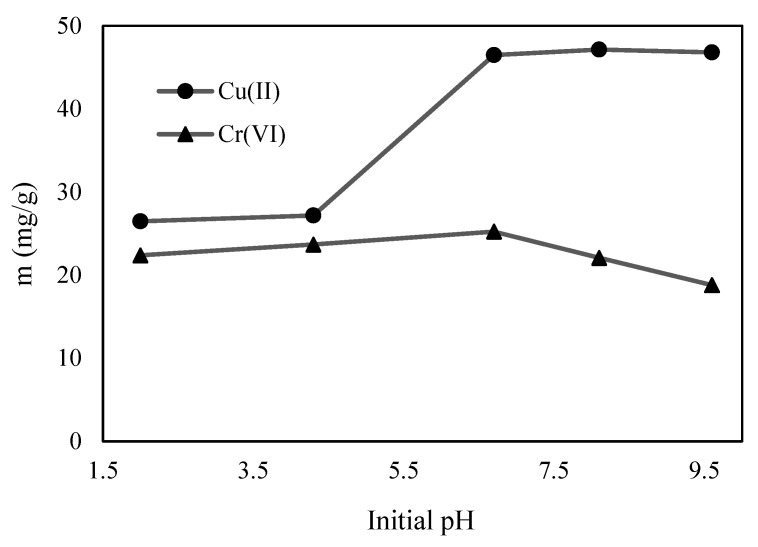
Effect of initial pH on Cu(II) and Cr(VI) adsorption. (C-ZLCH: 0.01 g, initial metal ion: 10 mg/L, volume (mL): 50, temperature: 30 °C, and time: 30 min).

The pH is the most critical parameter influencing the adsorbate (metal ions) and adsorbent (C-ZLCH) surface charges on Cu(II) and Cr(VI) adsorption [34]. The effect of pH on Cu(II) and Cr(VI) ion adsorption by C-ZLCH was examined by varying the pH from 2 to 9.6 (Figure 3). The Cu(II) adsorption increased from pH 4.3 to 6.7, reached a peak at pH 8.1, and then decreased slightly to pH 9.6. Negatively charged C-ZLCH surfaces enhanced the removal of Cu(II) via electrostatic interactions. Furthermore, Al^3+^ interacts with hydroxyl compounds under alkaline conditions, which is responsible for the cationic exchange between the zeolite and Cu(II) ions in solution [35,36]. Reaction mechanisms in Equation (3).

(3) $$3{\mathrm{Cu}\left(\mathrm{OH}\right)}_{2}+2\mathrm{Al}\;\to 3\mathrm{Cu}+2{\mathrm{Al}\left(\mathrm{OH}\right)}_{3}$$

Cr(VI) removal peaked at pH 6.7 before dropping to pH 9.6. One possible reason is that under acidic conditions, the hydrogen molecules (H^+^) in the solution can dissolve –NH_2_ to form NH_3_ on the surface of C-ZLCH, which increases the exposure of the particle surface active site, thus improving the removal efficiency of Cr(VI). Under alkaline conditions, there is competition between hydroxyl -OH- and Cr(VI) species (Cr_2_O_7_^2−^), thus decreasing the adsorption capacities [37,38]. Our findings for Cu(II) and Cr(VI) removal were comparable to those in [39,40,41,42].

### 3.4. Initial Concentration Effect

**Figure 4:**
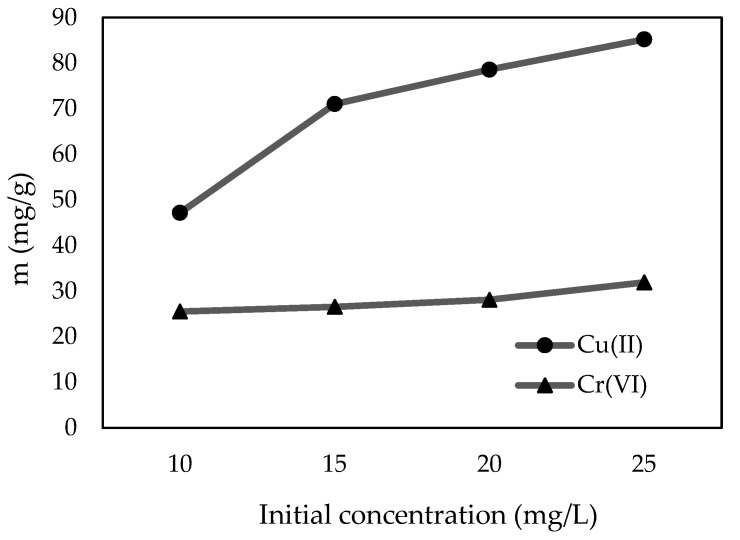
Effect of initial concentration on metal ions adsorption. (C-ZLCH: 0.01 g, volume (mL): 50, pH: 8.1 for Cu(II), pH: 6.7 for Cr(VI), temperature: 30 °C, and time: 30 min).

The influence of initial Cu(II) and Cr(VI) concentrations was investigated in the 10–25 mg/L range, 0.01 g/50 mL of C-ZLCH adsorbent, at pH 8.1 and 6.7, respectively. Figure 4 demonstrates that Cu(II) and Cr(VI) adsorption capacities increased from 47.15 to 85.23 mg/g and 25.53 to 31.93 mg/g, respectively. This is because of the high driving force of metal ions and the increase in the number of molecules, which enhances the amount adsorbed onto C-ZLCH [43,44,45,46,47].

### 3.5. Adsorption Isotherm Studies

Adsorption isotherms are essential for describing the adsorbent capability and interaction between the adsorbate (metal ions) and adsorbent (C-ZLCH). The obtained isotherm parameters are helpful for the proper analysis and design of adsorption systems. The experiment was conducted as follows: 0.01 g was mixed with 50 mL of the metal ion at different initial concentrations, ranging from 10–25 mg/L. The equilibrium concentration of each metal ion was calculated, and the corresponding equilibrium adsorption capacities were obtained. To explore the adsorption process, experimental equilibrium data were evaluated using different isotherm models, including the Langmuir (Equation (4)) and Freundlich (Equation (6)) models [25,40,41,42,43,44,45].

(4) $$m_{e}/m=(\frac{m_{e}}{m_{\max}})+1/(K_{1}m_{\max})$$

m is the amount of the adsorbent (mg/g),
K_l_ is the equilibrium constant of adsorption (L/mg),
m_max_ is the maximal adsorption capacities (mg/g), and
m_e_ is the equilibrium concentration (mg/L).

The essential characteristics of the Langmuir isotherm may be represented in terms of equilibrium, a dimensionless constant also known as the separation factor (R_L_) in Equation (4).

(5) $$R_{L}=(\frac{1}{1+{\mathrm{bm}}_{o}})$$

m_o_ is the initial concentration (mg/L), and
R_L_ is the separation factor, indicating the adsorption is either 0 < R_L_ (favorable), R_L_ > 1 (unfavorable) and R_L_ = 1 (linear).

The Freundlich model assumes heterogeneous surfaces and multilayer sorption. The linear equation is as follows:

(6) $$\mathrm{Ln}\;m=\ln K_{f}+\frac{1}{n}\times \;\ln m_{e}$$

K_f_ is the adsorption capacities (mg/g).
1/*n* is the intensity of adsorption.

**Figure 5:**
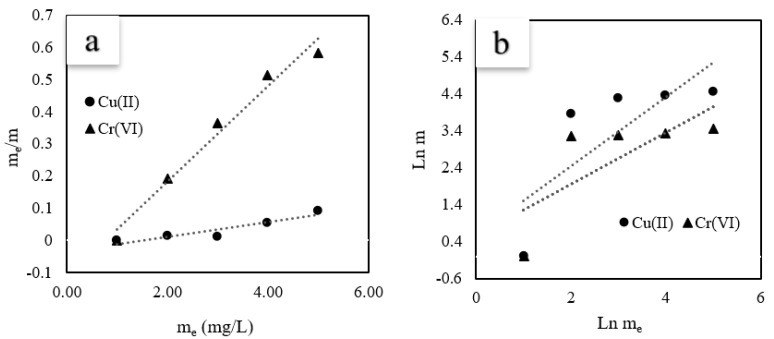
Linear curves of adsorption isotherm studies. (**a**): Langmuir, (**b**): Freundlich. (C-ZLCH: 0.01 g, volume (mL): 50, pH: 8.1 for Cu(II), 6.7 for Cr(VI), temperature: 30 °C, and time: 30 min).

The isotherm model curves, are shown in Figure 5, and the corresponding fitting results are listed in Table 1. Among the two models, the Langmuir model provided the best fit, because the linear correlation coefficients (R^2^) were 0.99 and 0.98 for Cu(II) and Cr(VI) adsorption, respectively. More importantly, the Langmuir and Freundlich parameters, R_L_ and 1/*n*, indicate a metal ion adsorption type of <1. These results suggest favorable Cu(II) and Cr(VI) adsorption.

### 3.6. Adsorption Kinetic Studies

**Figure 6:**
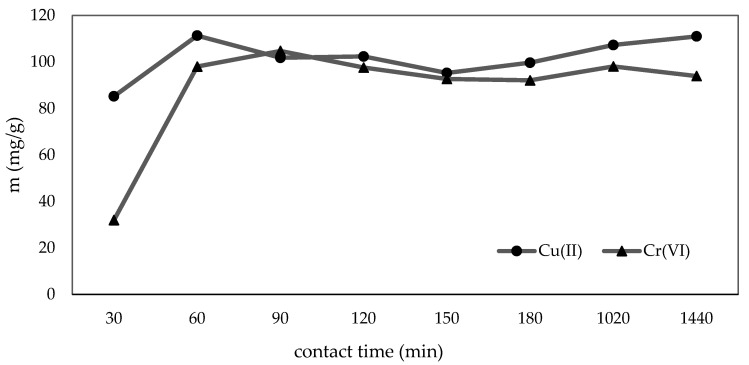
Effect of contact time on metal ions adsorption. (C-ZLCH: 0.01 g, volume: 50 mL, metal ions concentration: 25 mg/L, pH: 8.1 for Cu(II), 6.7 for Cr(VI), temperature: 30 °C, and time: 30 min).

The effect of time (30–1440 min) on the adsorption capacities of Cu(II) and Cr(VI) was studied. Figure 6 shows that the adsorption capacities of Cu(II) and Cr(VI) increased from 30 to 60 min and then gradually increased up to 90 min for Cr(VI). This is because C-ZLCH contains abundant functional groups (amino, methyl, and hydroxyl groups) and active sites, leading to the rapid adsorption of Cu(II) and Cr(VI). Subsequently, the adsorption capacities decreased and increased for both Cu(II) and Cr(VI) because the adsorption or desorption of metal ions occurred, leading to the reaction [48]. Finally, Cu(II) and Cr(VI) adsorption reached equilibrium at 60 and 90 min with adsorption capacities of 111.35 and 104.75 mg/g, respectively.

Adsorption kinetic studies are indispensable because they can provide information regarding the adsorption mechanism, which is essential for describing process efficiency [25,49,50]. This study used two kinetic models: pseudo-first order (Equation (7)) and pseudo-second order (Equation (8)). The linear form is calculated using the following equation:

(7) $$\log(m-\;m_{t})=\log m-K_{1}t$$

(8) $$t/m_{t}=1/(K_{2}m^{2})+t/m$$

where

K_1_ is the rate constant of pseudo-first-order kinetic model (min^−1^), and
K_2_ is the rate constant of pseudo-second-order kinetic model (g/mg min^−1^)
t is the time (min).

**Figure 7:**
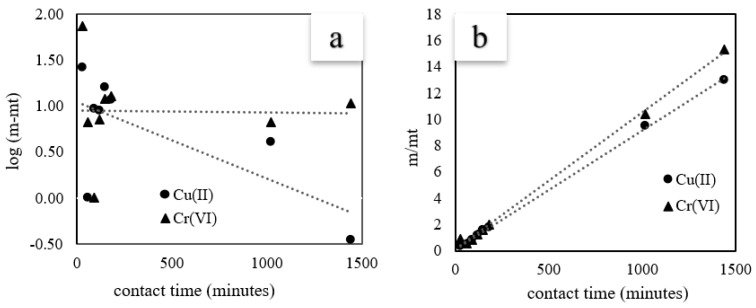
Linear curves of adsorption kinetic studies. (**a**): pseudo-first order, (**b**): pseudo-second order. (C-ZLCH: 0.01 g, volume: 50 mL, metal ions concentration: 25 mg/L, pH: 8.1 for Cu(II), 6.7 for Cr(VI), temperature: 30 °C).

The fitting curves of the isotherm models are shown in Figure 7 and the corresponding results are listed in Table 2. As can be seen that the linear correlation coefficient R^2^ value of the pseudo-second-order was better than the first-order model for both Cu(II) and Cr(VI). These results indicate that the adsorption rate is controlled by chemical reactions [39,51].

### 3.7. Effect of the Presence of Chemical Substances

**Figure 8:**
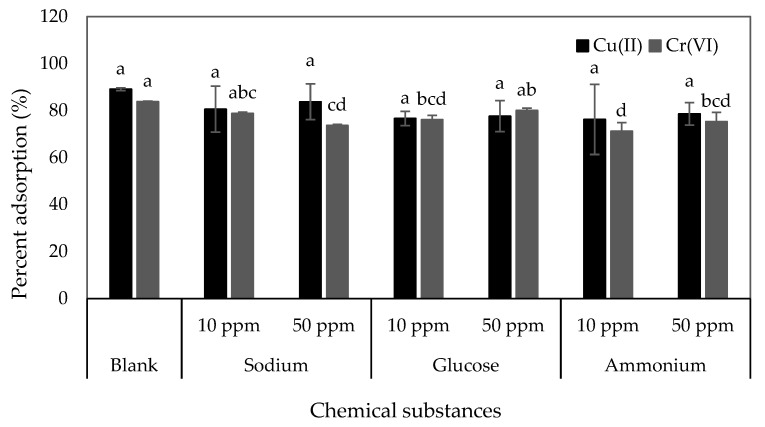
Effect of chemical substances on Cu(II) and Cr(VI) adsorption. [Blank: no presence of chemical substances, C-ZLCH: 0.01 g, volume: 50 mL, metal ions concentration: 25 mg/L, pH: 8.1 for Cu(II) at 60 min, pH: 6.7 for Cr(VI) at 90 min, temperature: 30 °C].

Many organic and inorganic compounds can affect the adsorption percentage of Cu(II) and Cr(VI) in the environment [52]. This study investigated the effects of Na^+^, glucose, and NH_4_^+^ at 10 mg/L and 50 mg/L on Cu(II) and Cr(VI) adsorption by C-ZLCH, as shown in Figure 8. Statistical analysis showed that the effect of chemical substances on Cu(II) removal was insignificant, in contrast to Cr(VI) removal. This suggests that Cr(VI) ions compete for binding sites on C-ZLCH [53]. 

### 3.8. Effect of the Presence of AR88 Dye

**Figure 9:**
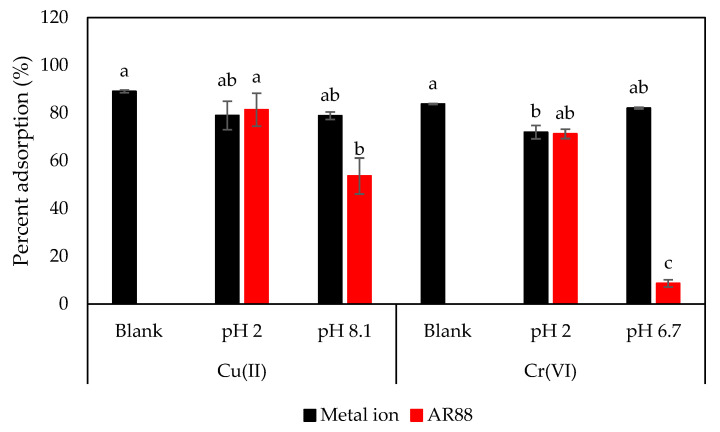
Effect of AR88 on Cu(II) and Cr(VI) adsorption. [Blank: no presence of AR88 dye, pH 8.1 for Cu(II) and pH 6.7 for Cr(VI), C-ZLCH: 0.01 g, volume: 50 mL, metal ions concentration: 25 mg/L, pH: 8.1 for Cu(II) at 60 min, pH: 6.7 for Cr(VI) at 90 min, temperature: 30 °C].

Industrial waste generally releases heavy metals and dyes, which are major industrial problems [54]. AR88 is a dye that harms humans and the environment, and is possibly trapped in soil [25]. In this study, we investigated the effect of AR88 on the simultaneous removal of metal ions and dyes (Figure 9). The experiment was conducted with optimal Cu(II) and Cr(VI) removal, initial AR88 of 10 mg/L, initial pH 2 and pH 8.1 for Cu(II), and pH 2 and pH 6.7 for Cr(VI). Statistical analysis showed that the presence of AR88 dye had no significant effect on Cu(II) removal. However, it significantly affected Cr(VI) removal at pH 2 compared to the absence of AR88 (blank). This indicates that the highest percentage of adsorption of Cu(II) and Cr(VI) was similar to that in the absence of AR88 dye (Figure 3). Moreover, the highest AR88 removal was observed at pH 2 for both Cu(II) and Cr(VI), similar to the results reported by Hidayat et al. [25].

### 3.9. Comparison with Other Adsorbents

Table 3 lists the adsorption capacities of different adsorbents. Thus, C-ZLCH is a promising adsorbent for removing Cu(II) and Cr(VI) from water. 

## 4. Conclusions

A carbonized zeolite/chitosan (C-ZLCH) composite was prepared via pyrolysis to remove Cu(II) and Cr(VI) from water. The results indicated that the pH was optimal at pH 8.1 and 6.7 for Cu(II) and Cr(VI), respectively. Cu(II) and Cr(VI) adsorption capacities were 111.35 and 104.75 mg/g after 60 and 90 min, respectively. For both Cu(II) and Cr(VI), the adsorption isotherm followed the Langmuir model, which is favorable (RL and 1/*n* < 1). The Cu(II) and Cr(VI) kinetic models were fitted to a pseudo-second-order model, which indicated chemical sorption on the C-ZLCH adsorbent. Statistical analysis showed no significant effect on Cu(II) removal in the presence of sodium, in contrast to the presence of glucose, ammonium, and AR88. However, there was a significant effect on Cr(VI) removal in the presence of chemical substances and AR88 dye.

## Figures and Tables

**Table 1:** Isotherm model parameters for Cu(II) and Cr(VI) adsorption onto C-ZLCH.

Adsorption Isotherm	Isotherm Constant	Metal Ions	
		Cu(II)	Cr(VI)
Langmuir	m_max_	88.64	34.57
	K_L_	226.64	14.51
	R^2^	0.99	0.98
	R_L_	0.00004	0.00068
Freundlich	K_f_	13015.09	950.66
	1/*n*	0.17	0.15
	R^2^	0.70	0.79

**Table 2:** Kinetic model parameters for Cu(II) and Cr(VI) adsorption onto C-ZLCH.

Adsorption Kinetic	Parameters	Metal Ion	
		Cu(II)	Cr(VI)
First order	m_e_	2.83	2.60
	K_1_	−0.0000133	−0.0000002
	R^2^	0.4715	0.0003
Second order	m_e_	111.11	96.15
	K_2_	0.0007	0.0011
	R^2^	0.9995	0.9977

**Table 3:** Comparison with other adsorbents.

Adsorbents	m (mg/g)		References
	Cu(II)	Cr(VI)	
Hematite Fe-oxide-coated sand (pH 1)	3.93		[55]
Chitin	2.80		[56]
Magnetite nano-adsorbent (MNA)	4.42		[57]
Fe_2_O_3_-carbon foam	3.8	6.7	[58]
HKUST-1/SiO_2_		62.38	[7]
NH_2_–ASNs		34.0	[59]
NH_2_–MSNs	53.5	42.2	[59]
Fly ash-derived zeolite	92.59		[60]
Fly ash-based zeolite A			[61]
Natural zeolite-coated magnetite		43.93	[62]
Amine-functionalized zeolite		13.5	[63]
Carbonized zeolite/chitosan	111.35	104.75	This study

## Data Availability

Not applicable.
